# Probing the role of intercalating protein sidechains for kink formation in DNA

**DOI:** 10.1371/journal.pone.0192605

**Published:** 2018-02-12

**Authors:** Achim Sandmann, Heinrich Sticht

**Affiliations:** Bioinformatik, Institut für Biochemie, Friedrich-Alexander-Universität Erlangen-Nürnberg, Erlangen, Germany; Wake Forest University, UNITED STATES

## Abstract

Protein binding can induce DNA kinks, which are for example important to enhance the specificity of the interaction and to facilitate the assembly of multi protein complexes. The respective proteins frequently exhibit amino acid sidechains that intercalate between the DNA base steps at the site of the kink. However, on a molecular level there is only little information available about the role of individual sidechains for kink formation. To unravel structural principles of protein-induced DNA kinking we have performed molecular dynamics (MD) simulations of five complexes that varied in their architecture, function, and identity of intercalated residues. Simulations were performed for the DNA complexes of wildtype proteins (Sac7d, Sox-4, CcpA, TFAM, TBP) and for mutants, in which the intercalating residues were individually or combined replaced by alanine. The work revealed that for systems with multiple intercalated residues, not all of them are necessarily required for kink formation. In some complexes (Sox-4, TBP), one of the residues proved to be essential for kink formation, whereas the second residue has only a very small effect on the magnitude of the kink. In other systems (e.g. Sac7d) each of the intercalated residues proved to be individually capable of conferring a strong kink suggesting a partially redundant role of the intercalating residues. Mutation of the key residues responsible for kinking either resulted in stable complexes with reduced kink angles or caused conformational instability as evidenced by a shift of the kink to an adjacent base step. Thus, MD simulations can help to identify the role of individual inserted residues for kinking, which is not readily apparent from an inspection of the static structures. This information might be helpful for understanding protein-DNA interactions in more detail and for designing proteins with altered DNA binding properties in the future.

## Introduction

The conformational plasticity of DNA is important for essential biological functions like transcription or replication [1, 2]. Deformation of DNA double strands can be facilitated by DNA sequence composition, environmental factors, or by the interaction with proteins [3, 4]. The following introduction will give a brief overview of some global and local types of DNA deformation, which are either observed in free DNA or induced by protein binding. The focus will be on protein-induced DNA kinks, which are the subject of the present study.

Even in the absence of protein, DNA can adopt different polymorphic forms, mainly B-DNA, A-DNA and Z-DNA. The most prevalent form is B-DNA since it is stable under aqueous conditions typical for living cells [5]. It is a right handed double helix with bases pointing perpendicular to the helical axis. It has a wide shallow major and narrow deep minor groove [6]. A-DNA is rather favoured under dehydrated conditions [7] and is a right-handed double helix with a narrow deep major and wide shallow minor groove. This inversion of groove width and depth is realized by an inclination [8] (rotation of successive base pairs along the short base pair axis with respect to the global helical axis) of the base pairs by ~ 20° [6]. In both cases the narrower groove has a more negative electrostatic potential [9–11]. Z-DNA is mainly observed under high salt concentrations [12, 13] and is a left handed double helix with a zigzag shaped backbone conformation. Apart from the solvent conditions, the DNA sequence itself also affects the preference for different DNA forms [12–14]. Another type of structural plasticity is the formation of a slight bent of the helical axis over a range of several base pairs. This deformation is not known to be favoured by effects from the medium but it is observed for A-tract sequences. A-tracts are defined as stretches containing only A:T base pairs and lacking TA base pair steps [15–17].

In addition to global deformations described above, there exist also local deformations, which are normally described in terms of deviation from the ideal B-DNA shape. One frequent type of local deformation is the formation of a kink in between two successive base pairs. This kind of deformation is characterized by an opening towards one of the grooves, resulting in increased roll angles [8, 18]. When DNA is constrained to a bend shape, for example when it forms small circles, local kinks can form spontaneously even in the absence of protein [19]. Interestingly, for strong bends, individual kinks can be more stable than an even distribution of the deformation over all base pair steps [20]. In addition, pyrimidine-purine steps have been shown to be easier deformed towards a kinked structure [6, 21].

However, kinks can also be induced by protein binding and are important for several biological functions. For example, long linear DNA molecules have to be deformed and compacted to fit into globular compartments like bacterial cells, cell nuclei or mitochondria. Chromosomal proteins like the histone complex help to realize this by facilitating kinking and supercoiling of the helical axis and DNA is often bound by a high number of chromosomal proteins [22–24]. Another situation, in which DNA deformation plays a role, is the recognition of DNA by transcription factors (TF). Kinking by transcription factors was shown to enhance the specificity of the interaction and to facilitate the assembly of multi protein complexes [25, 26].

Proteins that induce DNA kinks frequently exhibit amino acid sidechains that intercalate between the DNA base steps at the site of the kink. However, the role of intercalating residues may be strikingly different even for closely related proteins as exemplified by the crenarchaeal chromatin proteins Sac7d and Cren7 [27, 28]. In Sac7d the concomitant mutation of both intercalating residues to alanine leads to a drastically reduced kinking angle [29], whereas Cren7 contains only one intercalating residue, which can be replaced by alanine without significantly reducing the kink [30]. This example demonstrates that the role of intercalating residues for kink formation can hardly be assessed from an analysis of static wildtype structures alone. For most other systems with intercalating residues, there is yet little or no information about the role of individual sidechains for kink formation. This lack of data also hampers a comparison of different systems to unravel common principles of protein-induced DNA kinking.

To address this issue, we investigated in the present study (1) whether the intercalation of side chains is required for strong kinks, (2) whether the type of intercalated residues varies between different proteins and (3) whether multiple intercalated residues within the same kink vary in their relative importance. To this end we established a workflow to identify protein-DNA complex structures containing kinked DNA resulting in 88 complexes for 15 different systems. The complexes were analyzed with respect to their geometric properties and the type of intercalating amino acids. We then conducted molecular dynamics (MD) simulations of five complexes that varied in their architecture, function, and identity of intercalated residues. By comparative MD simulations of wildtype and mutant complexes, we assessed the role of individual intercalating sidechains for the maintenance of DNA kinks and finally compared the properties of the different complexes. This data also provides a basis for further experimental investigations of the respective systems.

## Methods

**Fig 1:**
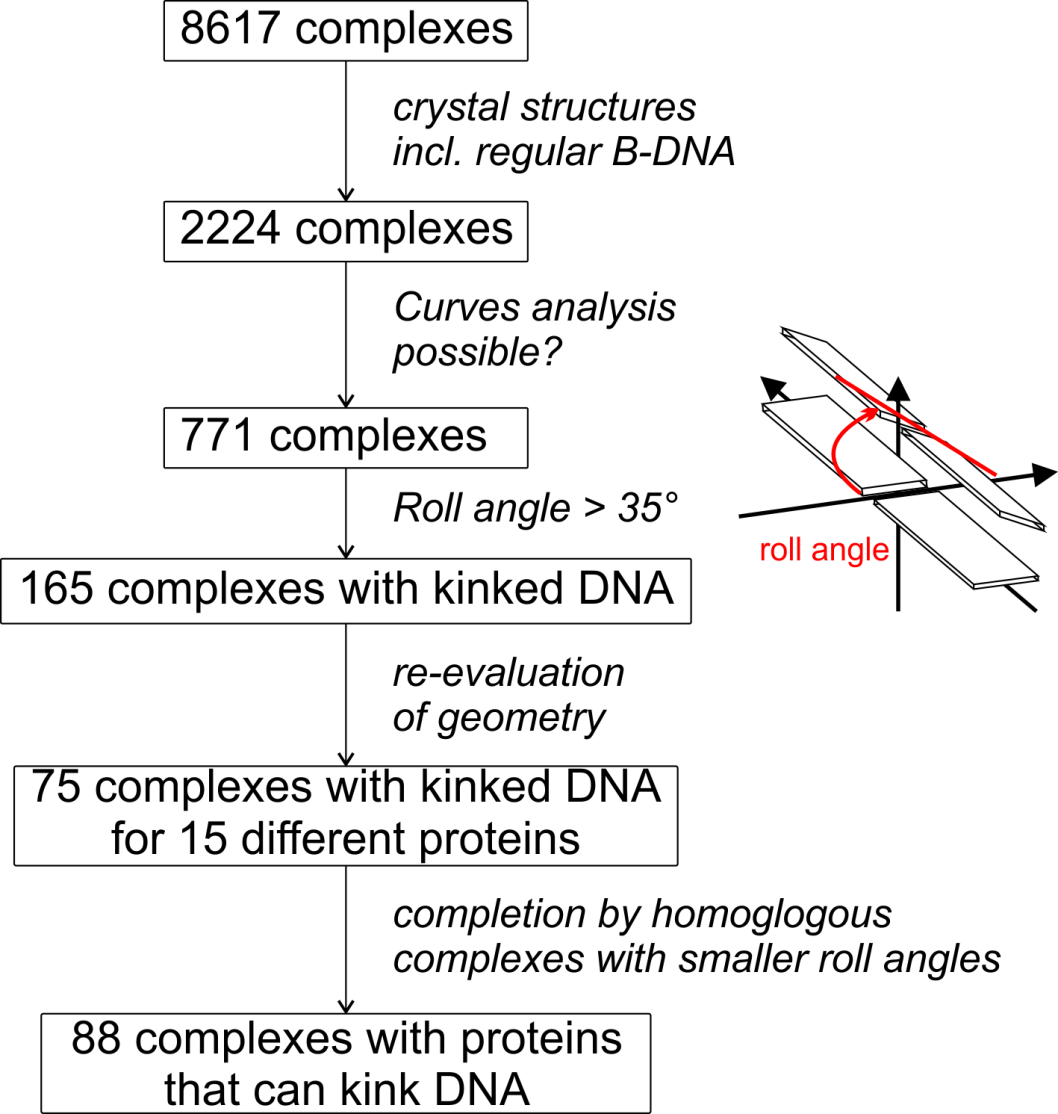
Flowchart of the approach to identify kinked DNA-protein complexes. Schematic presentation of the different steps taken to retrieve a set of 88 crystal structures of DNA-complexes for 15 different proteins from a total of 8617 structures available in the nucleic acid database NDB (see text for details of the procedure). The definition of the roll angle between two adjacent base pairs (red lines) is schematically depicted on the right.

Kinked protein-DNA complexes were identified from an analysis of the Nucleic Acid Database (NDB) [31, 32] containing a total of 8617 protein DNA complexes (accessed June 2016). Only crystal structures containing B-DNA segments were considered for further analysis of their roll angles using Curves+ [33]. The strategy, which is described in detail in the results section and in Fig 1, resulted in a total of 88 kinked DNA-structures for 15 different types of protein-DNA complexes.

From these 15 systems, five representative systems with intercalating protein side chains were selected for further analysis by molecular dynamics (MD) simulations. Simulations of the wildtype systems started from the complex crystal structures summarized in Table 1. For TFAM and TBP a few (< 10) terminal amino acids, which were lacking in the respective crystal structures, were added to obtain the physiological protein termini. For the remaining systems, N- and C-termini of the structure were capped with ACE or NME respectively to minimize possible effects of artificial charge. DNA strands were manually elongated by up to 5 base pairs on each side to enhance the overall conformational stability of the B-DNA geometry.

**Table 1:** List of all simulated systems including short names used in this work, crystal structure source and size of the simulated systems.

Protein	Short name	PDB ID	amino acid sequence	# of nucleic acid base pairs	# of Na^+^ Ions
**Sac7d**	Sac7d	1AZP[28]	M1-K66	11	14
**HMG domain of Sox-4**	Sox-4	3U2B[34]	G57-K133	16	21
**CcpA homodimer (one HPr bound per monomer)**	CcpA	3OQM[35]	M2-K333 + A2-E88	16	64
**Complete Transcription factor A, mitochondrial (TFAM)**	full length TFAM	3TMM[36]	S43-C246	30	43
**HMG-Box-2 of TFAM**	TFAM	3TMM[36]	G152-C246	15	25
**TATA-Box Binding Protein**	TBP	1CDW[37]	S158-T339	16	15

Alanine and glycine mutants were generated from the final wildtype setup by manual truncation of the intercalating sidechains. The Sox-4 A67M mutant was generated from the final structure of the M67A mutant simulation (run 2) by selecting an appropriate rotamer of the methionine side chain and adjusting the chi-angle of the adjacent base Ade9 (chain B) by 32° to minimize clashes. For TFAM, the complete protein, as well as the isolated HMG-Box-2, was simulated in complex with DNA.

All MD simulations performed with AMBER [38–40]. According to an established strategy for protein-DNA complexes [41–43] all systems were neutralized by adding an appropriate amount of sodium counter ions resulting in a final ionic strength of 70–130 mM depending on the system. Each system was placed in a periodic TIP3P water box [44] extending at least 12 Å in all directions from the solute. The ff99bsc0 force field [45] was used. Long-range electrostatics were calculated with the particle mesh Ewald (PME) approximation [46]; Shake was used to constrain hydrogen atoms during equilibration and simulation [47]. Minimization, equilibration and the MD production phase were carried out with pmemd. Minimization was run for 10,000 steps and switched from steepest descent to conjugate gradient after 500 cycles. Equilibration was run for 40,000 steps / 80 ps. The systems were heated from 30 K to 100, 200 and 300 K in three 20 ps runs with backbone restraints of 2.0 kcal·mol^-1^·Å^-2^ and relaxed at 300 K with backbone restraints of 0.2 kcal·mol^-1^·Å^-2^ for another 20 ps. Each system was minimized once and equilibrated for each run. Random velocity assignment from a Boltzmann distribution for 30 K as starting point resulted in different sampling for each run. Production runs were generated at 300K using the weak-coupling algorithm [48] and a Berendsen barostat in an NTP ensemble. The only restraints used in the production phase were applied to the hydrogen bonds of the terminal base pairs to avoid melting of the short DNA segments. When exceeding 2.3 Å, the length of these hydrogen bonds was restrained by a harmonic potential (50.0 kcal·mol^-1^·Å^-2^). At least two independent runs were conducted for each system (simulation time 250–500 ns depending on the conformational properties of the system). AMBER cpptraj [49] and Curves+ [33] were used for evaluation. Roll angles were analyzed with Curves+ based on 2000 equidistant snapshots extracted from the MD simulation. Chimera [50] was used for visualization, and plotting was done with gnuplot [51].

## Results and discussion

### Identification of kinked DNA-protein complexes

The aim of the present work was the structural analysis of kinked DNA-protein complexes with intercalated side chains. To obtain a sufficiently large set of such complexes, we parsed the nucleic acid database (NDB), which contains 8617 entries of nucleic acids in complex with proteins (Fig 1). Since we were interested in kinked B-DNA segments, entries containing RNA, modified DNA, or single stranded DNA were omitted. In addition, we did not consider structures determined by NMR, which frequently exhibit a lower resolution compared to crystal structures.

The remaining 2224 complexes were further analyzed with Curves+ [33] in a scripted process to obtain the geometric parameters of the DNA. This automated procedure succeeded for 771 complexes, but produces errors for the remaining complexes. A manual inspection revealed that these errors occurred e.g. for structures with multiple separate DNA double strands within one crystal unit-cell or a different number of bases in each of the paring strands. Of the 771 structures producing an output for the Curves+ analysis, 165 contained at least one roll angle larger than 35° indicating a kink or bend in the DNA geometry (Fig 1). We preferred to analyze individual roll angles instead of the overall kink, because we aimed to identify systems with defined sharp kinks instead of systems containing gradual bends that include more than one base pair.

The 165 structures were manually checked and edited if necessary. For example, in some cases dangling single-stranded ends on the B-DNA caused Curves+ to shift the register of paired bases by the number of dangling nucleotides, which was corrected by removing the dangling ends. Some complexes were discarded, because the large roll angles resulted from base flip outs and not from distortions of paired base pairs, which are the focus of the present study. In addition, we excluded in this phase the large number of nucleosome structures, which are non-intercalating and exhibit a large heterogeneity regarding the position and sequence of the kinks. The filtering and editing of the identified complexes confirmed the presence of large kinks (> 35°) in 75 DNA stretches, which interact with 15 different proteins. To complete the dataset, the databases Uniprot and PDB were searched for other protein-DNA complexes containing these 15 proteins. This procedure allowed to identify 13 additional homologous complexes, which were not detected in the original analysis, because the largest kink angle is < 35°. These complexes are nevertheless interesting for further analysis because they give information about the range of kink angles induced by the same protein.

The overall procedure described above also demonstrated that an automated analysis of DNAs using Curves+ is not trivial and may produce errors and artefacts in different states of the analysis. To avoid the inclusion of wrong candidates in our dataset, we discarded complexes with error messages in the Curves+ analysis completely. In addition, all complexes included in the final dataset were manually corrected and re-evaluated to avoid a misinterpretation of kink angles (e.g. due to dangling ends; see above). We are aware that our approach may fail to detect some systems with large kink angles due to inherent problems of an automated geometry analysis. However, the resulting dataset of 88 non-covalent complexes for 15 different proteins still remains sufficiently large for the subsequent structural analysis and has the advantage of containing no false-positive hits. The systems identified and the respective PDB codes are summarized in S1 Table.

**Fig 2:**
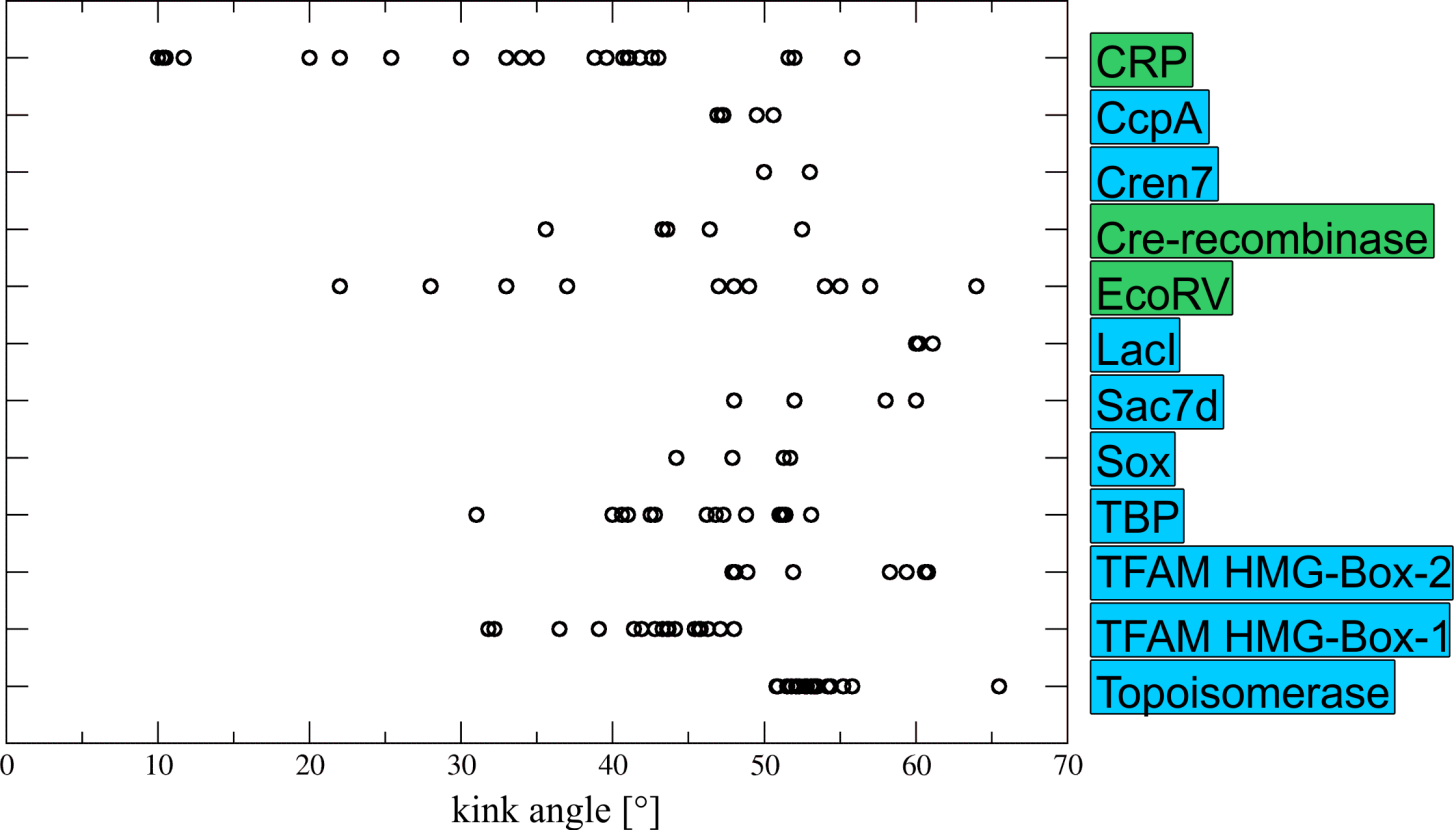
Distribution of kink angles for different systems. Multiple dots in a single line result from the analysis of multiple DNA-complexes available for the same protein. Proteins in green boxes have no intercalating side chains, whereas proteins in blue boxes do have intercalating side chains. Values of the kinks produced by TFAM HMG-Box-1 and HMG-Box-2 are depicted separately.

In 11 of the 15 systems, protein side chains are intercalated between base pair steps at the kinked position. This shows that intercalation is common, but not essential for kink formation. In case that multiple DNA-complex structures were available for the same protein, we noted that systems without sidechain intercalation show a rather broad distribution. This becomes particularly evident for the CRP-DNA complexes showing kink angles in the range from 10° to 55° (Fig 2; we henceforth use the term kink angle to describe the largest roll angle detected in a DNA-protein complex). The same trend is also visible for the non-intercalating complexes formed by EcoRV and Cre-recombinase. In contrast, complexes with intercalating side chains frequently show a narrower distribution of kink angles suggesting that insertion may be required to stabilize a particular kink angle.

To investigate the role of intercalating residues on DNA geometry in more detail, we conducted MD simulations for five of the systems that have inserting side chains and mutants in which the respective side chains were replaced by alanine. The five proteins (Table 2) were chosen for diversity with regards to protein function (DNA compaction proteins and transcription factors), type of residue used in side chain insertion (Met, Val, Leu, Phe), number of inserted side chains (two or three), DNA selectivity at the kinked position (unselective vs. selective for particular base steps). For clarity, the results of the MD simulations will be first presented for each system separately followed by a final comparison of the properties detected.

**Table 2:** List of all simulated systems, the function of their protein part, types of intercalating residues and DNA selectivity at the kinked position. Residues in parenthesis are only partially intercalating.

Protein	Function	Intercalated residues	Kinked base step
**Sac7d**	DNA compaction	Met, Val	Any
**Sox-4**	Transcription factor (TF)	Met, Phe	TT
**CcpA**	TF (homodimer)	Leu, Leu	CG
**TFAM**	TF and DNA compaction	Leu, (Val, Phe)	CA
**TBP**	TF	Phe, Phe	TA and AG

### The Sac7d-DNA complex

**Fig 3:**
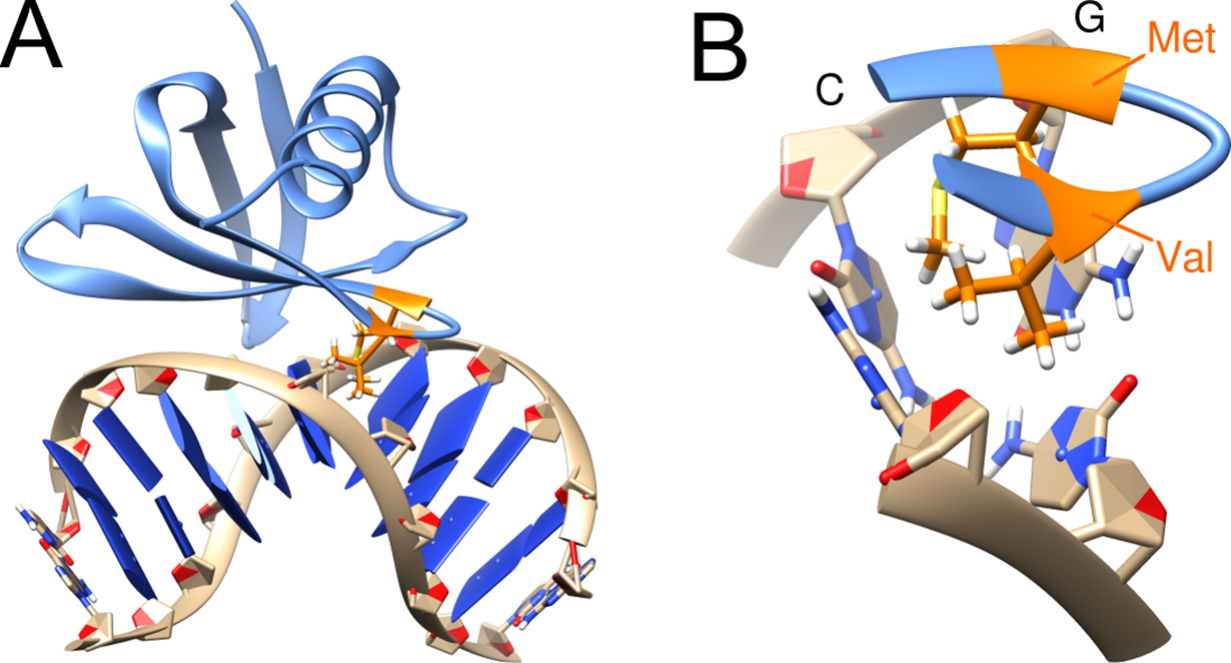
Structure of the Sac7d-DNA complex. (A) Structure of the Sac7d-DNA complex with intercalated residues highlighted in orange. (B) Enlargement showing the kinked DNA base pair step and intercalating residues Val26 and Met29. The kinked CG base step is also indicated.

Sac7d is a small and highly abundant chromosomal archaeal protein which facilitates DNA compaction by inducing one kink per protein in DNA strands. Sac7d binding is also important for stabilization of DNA at high temperatures [52]. It binds the minor groove of DNA at sequence nonspecific positions and intercalates the side chains of the hydrophobic residues V26 and M29 between the same base pair step (Fig 3).

**Fig 4:**
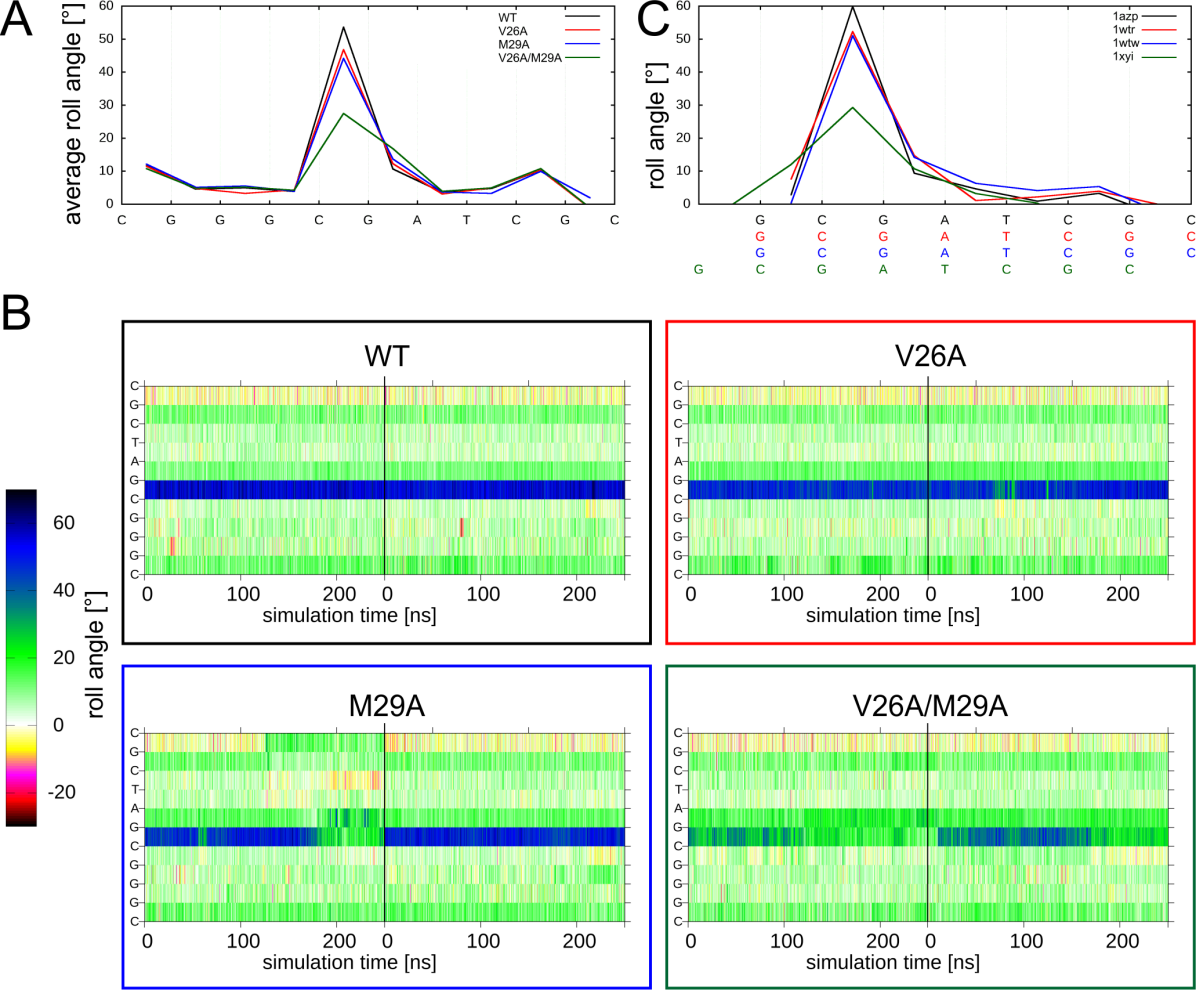
DNA deformation by wildtype and mutant Sac7d. (A) DNA roll angle profile of average values calculated from the MD simulations. (B) Time course of the roll angles for all base steps over the MD simulation. The individual bases are plotted as y-axis starting with the 5’-end at the bottom. The lowest row in the diagram thus represents the time course of the roll angle between C1 and G2. The vertical black line denotes the boundary between the two independent 250-ns MD simulations performed for each system, which are presented in a single panel. Colors of boxes match line colors of average roll angle plots of corresponding systems in (A). (C) DNA roll angle profile of the roll angles found in the crystal structures of the wildtype and mutant Sac7d proteins. The PDB codes of the respective complexes are indicated. Wildtype and mutants are color coded as in (A).

The average roll angles observed over the MD simulation time for the wild-type and mutant systems are shown in Fig 4A. The protein wild type–DNA system exhibits an average kink of 54°. For V26A and M29A alanine single mutants, the average kink value is slightly lowered to 47° and 44°, respectively. In simulations of the V26A/M29A double mutant, the average kink angle is significantly decreased to 28° (Fig 4A). The inspection of the roll angles over the simulation time (Fig 4B) reveals that the reduction of the average roll angles observed for the mutants is not accompanied by other larger-scale changes of the DNA geometry. In particular, the site of largest kink angle remains unaltered, which is different from some of the other systems described below.

For all three Sac7d mutants investigated in the present study, crystal structures are available [29], which allowed us to compare our simulated roll angles to those observed in the crystal structures. The values obtained from our simulations are in good agreement with the values detected in the respective crystals (Fig 4A and 4C) indicating that the changes in DNA kink angle upon protein mutation are adequately reproduced by our MD simulations.

Taken together, the investigations above suggest that both side chains have an at least partially redundant role for kink formation since either side chain can induce the major part of the deformation on its own (Fig 4A). However, the maximal kink angle is only observed for the wildtype with two intercalating side chains (Fig 4A).

### The Sox4-DNA complex

The Sox protein family are transcription factors relevant for embryonic development and cell differentiation [53]. Although the different members of this protein family are responsible for different developmental signals, the amino acid sequences, as well as the DNA binding motifs (TTGT) of the DNA binding HMG domain of those different proteins, are rather similar [54]. The HMG domain of Sox-4 investigated in the present study can thus be regarded as a representative case for the HMG boxes of the Sox family.

**Fig 5:**
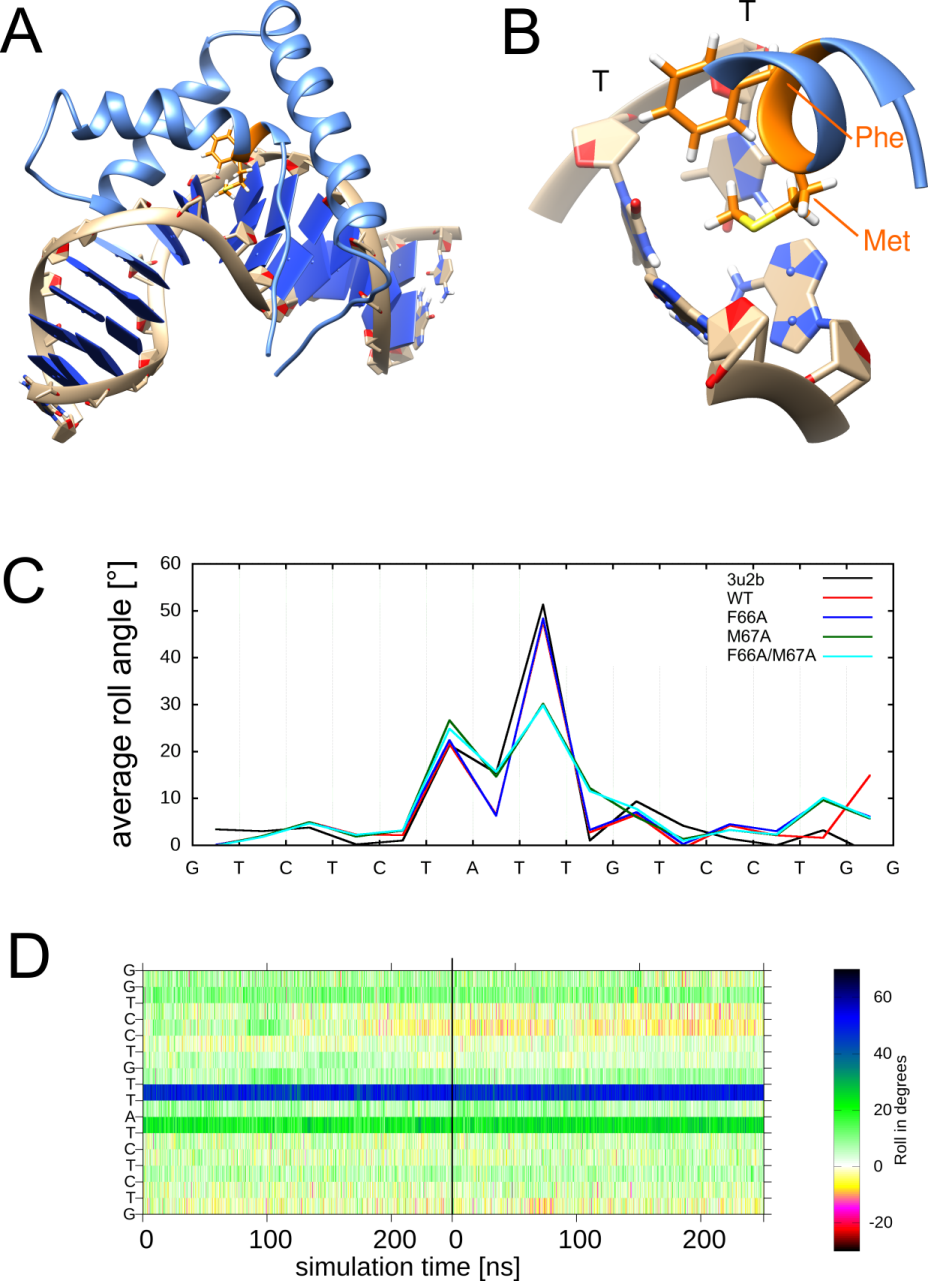
Structural properties of the Sox-4-DNA complex. (A) Structure of Sox-4-DNA complex with intercalated ‘FM wedge’ residues highlighted in orange. (B) Enlargement showing the kinked DNA base pair step and intercalating residues Phe66 and Met67. The kinked TT base step is also indicated. (C) DNA roll angle profile of Sox-4 in the simulations of wildtype and mutant Sox-4 proteins (the wildtype crystal structure 3u2b is shown as reference). (D) Time course of roll angles for Sox-4 A67M mutant. Values are shown for all base steps over the MD simulation. The vertical black line denotes the boundary between the two independent 250-ns MD simulations performed, which are presented in a single panel.

Sox-4 contains two intercalating residues, F66 and M67 (Fig 5A and 5B), which were termed the ‘FM wedge’ [34, 54]. The average kink angle of the wild type system is 48°, which is close to the 51° observed in the crystal structure. The F66A single mutant shows a similar average roll angle of 48°, whereas the M67A single mutant shows a reduced kink angle of 30° (Fig 5C, S1 Fig). This indicates that F66, although positioned directly at the kink site, is not important for maintenance of the kink. The double mutant F66A/M67A shows an almost identical roll angle profile than the M67A single mutant with an average roll angle of 30°, like the M67A single mutant (Fig 5C, S1 Fig). Thus, F66A does not affect the kink angle neither when mutated alone nor in the M67A background. Taken together, our results indicate that the two intercalating residues of the ‘FM wedge’, which were considered of similar importance from the static structure, actually play a clearly distinct role for kink formation. The importance of the methionine for kinking is also in line with a previous mutational study of a related HMGD domain, containing a Tyr-Met motif at the site of the kink [55]. Replacement of the methionine by alanine resulted in a reduced kink as demonstrated by crystallographic analysis [55]. However, to the best of our knowledge, such experimental information is not yet available for the remaining systems investigated below.

For all systems studied thus far, we noted that an adjustment of the kink angles as a response to mutation are rather fast and occur within a few nanoseconds. This prompted us to use Sox-4(M67A) as a model system to investigate the reversibility of the changes. For that purpose, we used the final structure of the Sox-4(M67A) simulation (S1 Fig; run2) that exhibits a reduced kink angle and reverted the sequence back to wildtype. The time course of the roll angles (Fig 5D) reveals that the large kink angle characteristic for the wildtype is already formed within the equilibration stage and the first few nanoseconds of the simulation. The overall conformational stability of the reverted Sox-4(A67M) mutant is similar to the one of the original wildtype simulation (Fig 5D, S1 Fig) indicating that the changes resulting from the M—> A mutation are reversible.

Another finding gained from the alanine mutations in Sox-4 was that the kink angle of ~30° observed in the M67A mutant cannot further be reduced by an additional F66A mutation (Fig 5C). This prompted us to investigate whether the remaining kink of ~30° is due to a residual intercalation formed by the A67 sidechain. For that purpose, we simulated a M67G single mutant, which lacks the sidechain methyl group (S2 Fig). Unexpectedly, the M67G mutant even retained a larger kink angle compared to the M67A variant. Although the exact structural feature responsible for this difference remains elusive, the properties of the M67G variant at least indicate that the remaining kink angle of ~30° is not due to a residual intercalation of the alanine sidechain.

We also investigated the potential role of an alanine sidechain in kink formation at a distinct site of the Sox-4 DNA complex. In addition to the major kink formed at a TT step, Sox-4 induces a second smaller kink of ~30° at an adjacent TA step (Fig 5C). Upon closer inspection of the structure, an alanine (A87) is found to be in contact with this TA base step. Therefore, we investigated an A87G variant of Sox-4, which proved to have no effect on the magnitude of the kink at the TA base step (S2 Fig). Taken together, the results for the M67A/M67G and A87G simulations suggest that the presence of a short alanine sidechain is not responsible for the residual kinks of ~30° observed in the absence of intercalating residues.

### The CcpA-DNA complex

**Fig 6:**
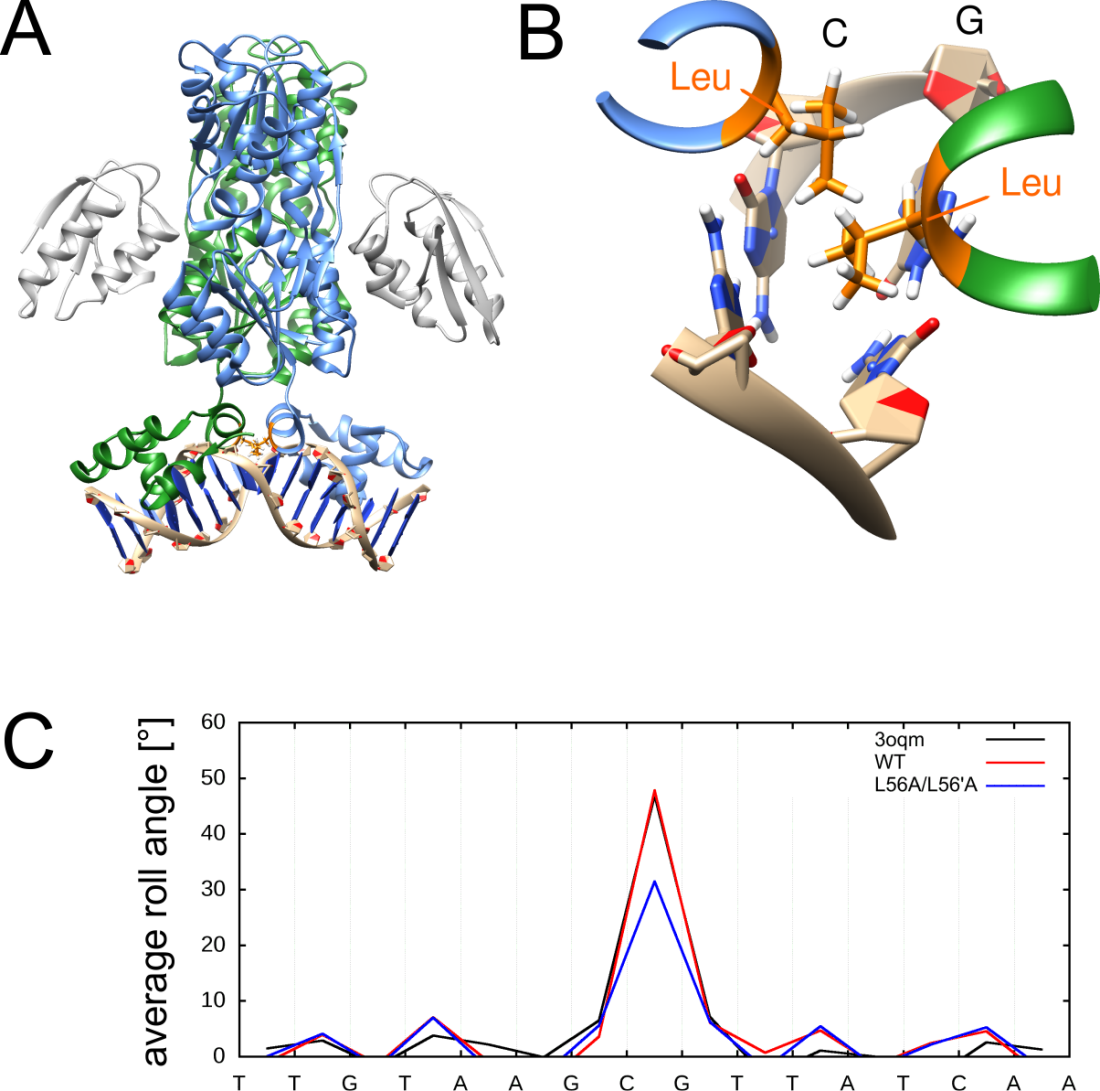
Structural properties of the CcpA-DNA complex. (A) Structure of CcpA-DNA complex with intercalated residues highlighted in orange. The two subunits of CcpA are colored in blue and green, respectively, and the corepressor HPr is shown in gray. (B) Enlargement showing the kinked DNA base pair step and intercalating residues L56 and L56’. The kinked CG base step is also indicated. (C) DNA roll angle profile of CcpA in the simulations of wildtype and mutant CcpA proteins (the wildtype crystal structure 3oqm is shown as reference).

CcpA is a homodimeric bacterial transcription factor (Fig 6A), which controls the carbon metabolism and exhibits structural similarities to the lac repressor (LacI). Both proteins contain two globular domains per subunit, which also form the dimer interface. Unlike LacI, CcpA requires a corepressor (Hpr) for efficient DNA binding [56, 57]. CcpA and LacI exhibit a helix-turn-helix-loop-helix motif which is connected to the protein core via a hinge helix. The latter helix of CcpA contains residues L56, which intercalates into the minor groove at the centre of the DNA sequence motif to induce a kink. Due to the dimeric nature of CcpA, two leucine sidechains, each originating from one subunit, intercalate at the same base step (Fig 6B).

The average kink angle of CcpA is 48°, which is consistent with the crystal structure (47°) (Fig 6C). The kink angle is reduced to 31° when the inserted L56 side chains of both monomers are mutated to alanine. This indicates the importance of L56 insertion for kink formation. Time dependent plots show stable roll angle patterns for wild type as well as mutated system. Apart from a reduced kink angle, we did not detect any larger conformational changes of the protein or DNA upon L56A mutation (S3 Fig).

### The TBP-DNA complex

**Fig 7:**
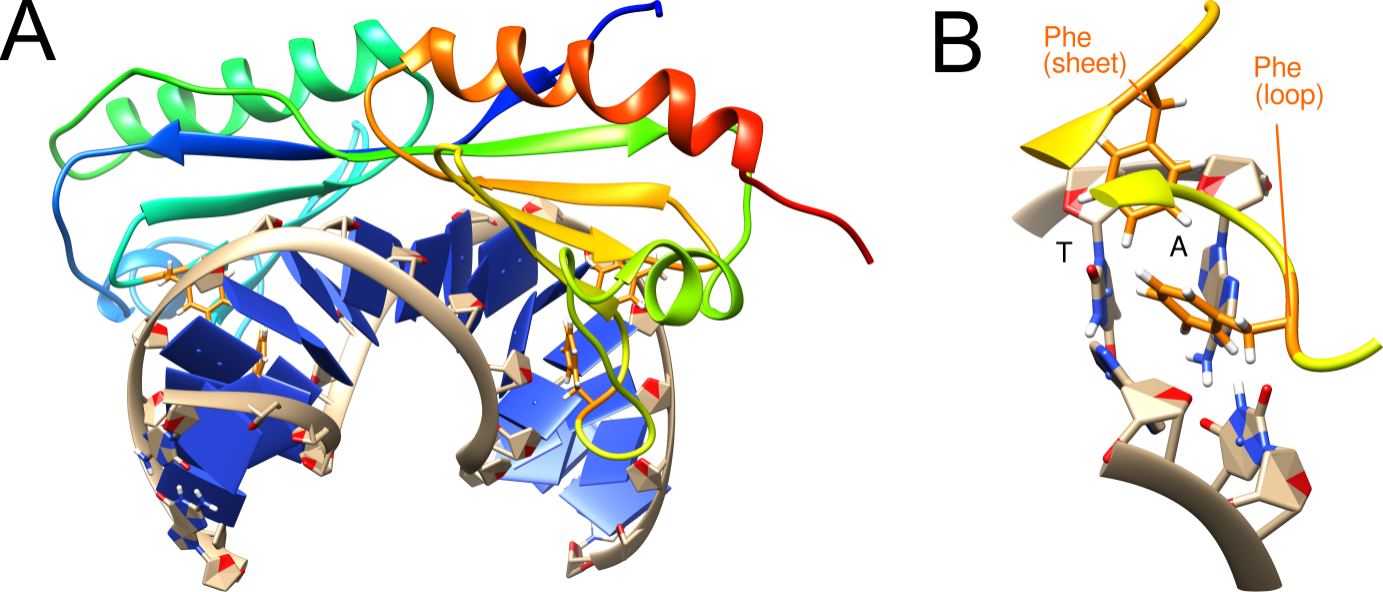
Structure of the TBP-DNA complex. (A) Structure of TBP-DNA complex with intercalated residue highlighted in orange. (B) Enlargement showing one of the kinked DNA base pair steps (TA) and the intercalating residues Phe284 (loop) and Phe301 (sheet).

The TATA Box binding protein TBP is a eukaryotic transcription factor that specifically recognizes a DNA sequence called TATA box [58]. The protein is formed by one chain, but has two almost C2-symmetric subdomains (Fig 7A). It consists largely of one large saddle-shaped beta-sheet that binds to the DNA minor groove with its concave side. It also contains one short helix and loop at each end of the saddle like structure and two long helices on the convex side that doesn’t face the DNA. At both ends of the saddle-like beta-sheet, two phenylalanine side chains are inserted into the DNA minor groove (F193, F210 and F284, F301). The two intercalating sidechains at each end can be further distinguished according to their position in the structure: One is located in the outermost β-strand and one in the adjacent loop. These residues were termed Phe(sheet) / Phe(loop) and were investigated separately in the present study (Fig 7B).

**Fig 8:**
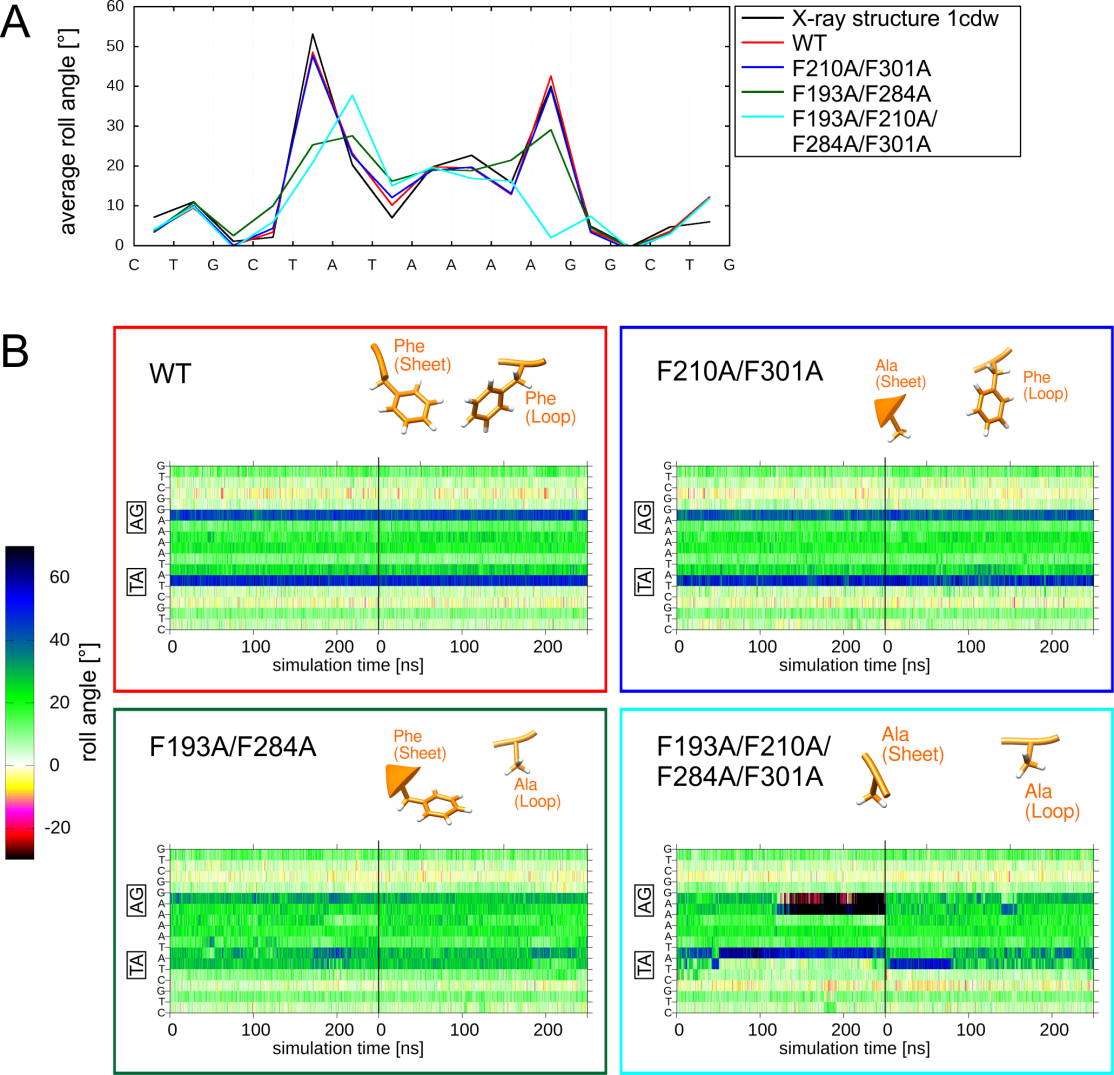
DNA deformation by wildtype and mutant TBP. (A) DNA roll angle profile of TBP in the simulations of wildtype and mutant TBP proteins (the wildtype crystal structure 1cdw is shown as reference). The DNA sequence is shown as x-axis indicating that there are two kinks formed between a TA and an AG base step, respectively. (B) Time course of the roll angles for all base steps over the MD simulation. The two base steps exhibiting the largest kinks are highlighted by boxes at the y-axis. The vertical black line denotes the boundary between the two independent 250-ns MD simulations performed for each system, which are presented in a single panel. Colors of boxes match line colors of average roll angle plots of corresponding systems in (A). The type of mutation present in the individual systems is structurally depicted at the top of each panel.

The roll angle profile of the wild type simulation shows two kinks of 49° and 43° at the intercalating positions, respectively (Fig 8A). Although the kinks are slightly (3–4°) larger in the crystal structure, the overall roll angle profile is highly similar to that of the wildtype simulation.

To investigate the role of the intercalating phenylalanines in detail, three sets of mutants were created, in which either the Phe(sheet) residues F210/F301 or the Phe(loop) residues F193/F284, or all four phenylalanines were mutated to alanine. When the Phe(sheet) residues are mutated (F210A/F301A), the kink values are only slightly reduced to 48° and 39° (Fig 8A). In contrast, mutation of the Phe(loop) residues (F193A/F284A) resulted in significantly reduced kink angles of 25° and 29°. When both sets of phenylalanines are mutated (F193A/F210A/F284A/F301A) the average roll angle profile is altered significantly. The first kink at the TA base step is shifted to the adjacent AT step, and the second kink at the AG step vanishes completely (Fig 8A). For this quadruple mutant, the time course of the roll angles (Fig 8B) confirms the conformational instability and reveals a mixture of structures with kinks at different positions, which is not readily evident form the average kink angle shown in Fig 8A. In contrast, the wildtype and double mutants exhibit a rather stable behaviour of the kinks over the simulations time (Fig 8B). This indicates that either Phe(sheet) or Phe(loop) are sufficient to confer conformational stability to the system. However, the role of both classes with respect to kink formation is different and Phe(loop) in contrast to Phe(sheet) is required to maintain the magnitude of the wildtype kink.

### The TFAM-DNA complex

**Fig 9:**
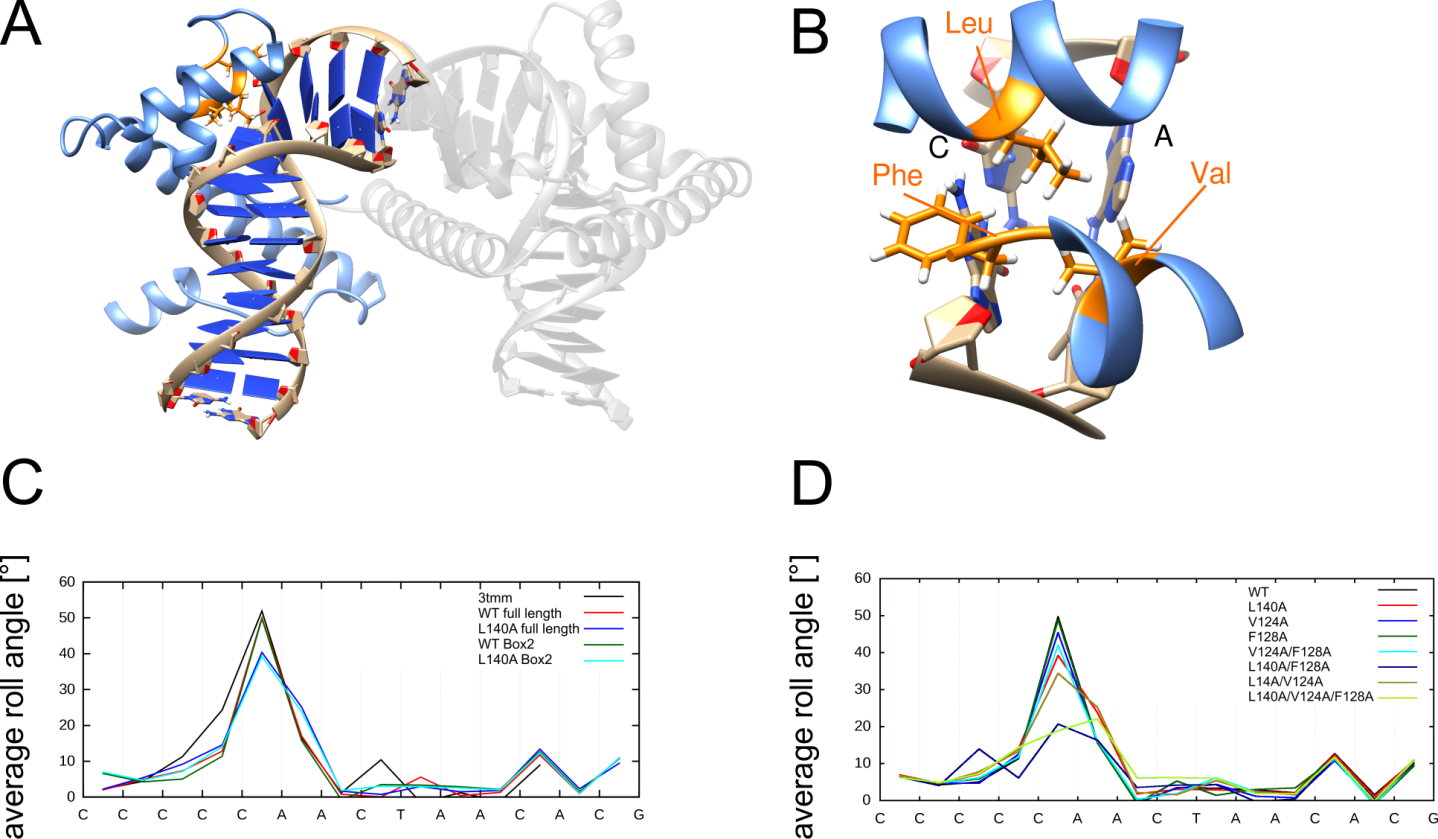
Structural properties of the TFAM-DNA complex. (A) Structure of the TFAM-DNA complex. HMG-Box-2 is highlighted in color and HMG-Box-1 is shown in grey. (B) Enlargement showing the kinked CA base step (HMG-Box-2). The intercalating L140 as well as the partially inserting V124 and F128 are shown in stick presentation. (C) DNA roll angle profile of TFAM for the simulations of full-length and truncated systems containing only Box-2. The wildtype crystal structure (3tmm) is shown as reference. (D) DNA roll angle profiles for different TFAM Box-2 mutants.

TFAM is a sequence specific mitochondrial transcription factor, which also induces strong compaction in DNA strands [59–61]. It consists of two DNA binding HMG-Boxes which are linked by one helix. Both HMG-Boxes bind the DNA at the minor groove and induce deformation of the DNA which results in two 90° bends of the DNA strand (Fig 9A). HMG-Box-1 induces a complex kink comprising two immediately adjacent base steps, whereas HMG-Box-2 induces a single kink at a CA base step. Due to this special situation in Box-1, we focused our analysis on Box-2 only. The major intercalating residue of Box-2 is L140, whereas V124 has been described previously as partially inserting [62]. From visual inspection, we classified F128 as an additional partially inserting residue (Fig 9B).

Before studying the individual role of these residues in detail, we first assessed the influence of Box-1 on kink formation by Box-2. For that purpose, we performed two sets of simulations either for a full-length system with both HMG-Boxes or for a truncated system containing only Box-2. Simulations were done for the wildtype and L140A mutants resulting in a total of four simulations (Fig 9C).

The average roll angle values for the kink induced by Box-2 are 50° in the full-length TFAM, as well as the truncated Box-2 segment (overlapping red and green lines in Fig 9C). This corresponds well with the X-ray structure that was used as starting point for the simulation (52°, black line in Fig 9C). For the L140A mutant of the entire TFAM protein, an average kink angle of 40° is found, while the truncated Box-2 system shows an average kink angle of 39° (blue and cyan lines in Fig 9C). This indicates that Box-1 has no significant remote effect on the properties of the kink induced by Box-2, neither for the wildtype nor for the L140A mutant. Based on this finding, all subsequent simulations were performed using Box-2 only.

Single mutants of the partially inserting residues (V124A and F128A) had a smaller influence on the average kink angle (45° and 49°, respectively) compared to the L140A variant (39°) (Fig 9D). However, although the L140A variant showed a slightly larger effect than mutants of the partially inserting residues, none of the single mutants resulted in a large reduction of the kink angle.

Investigation of double mutants revealed one particular combination (L140A/F128A) that lead to a drastically reduced kink angle of 22°, whereas the remaining double mutants showed a much smaller effect (Fig 9D). Thus, this analysis proved F128 to be more important for kink formation than V124, which is not readily apparent from an analysis of the static wildtype structure. The kink observed for the L140A/F128A variant cannot be further reduced by an additional V124A mutation. Instead, the L140A/V124A/F128A triple mutant exhibits an increased conformational heterogeneity and the kink becomes shifted from the CA to the adjacent AA base step (Fig 9D and S5 Fig). The observation that L140A or F128A mutants have only small effects, whereas a L140A/F128A double mutant exhibits a large effect, is similar to the situation of the V24A/M29A mutants in Sac7d (Fig 4), indicating a set of two key residues of which at least one is required to maintain the kink.

### Comparison of the different systems

**Fig 10:**
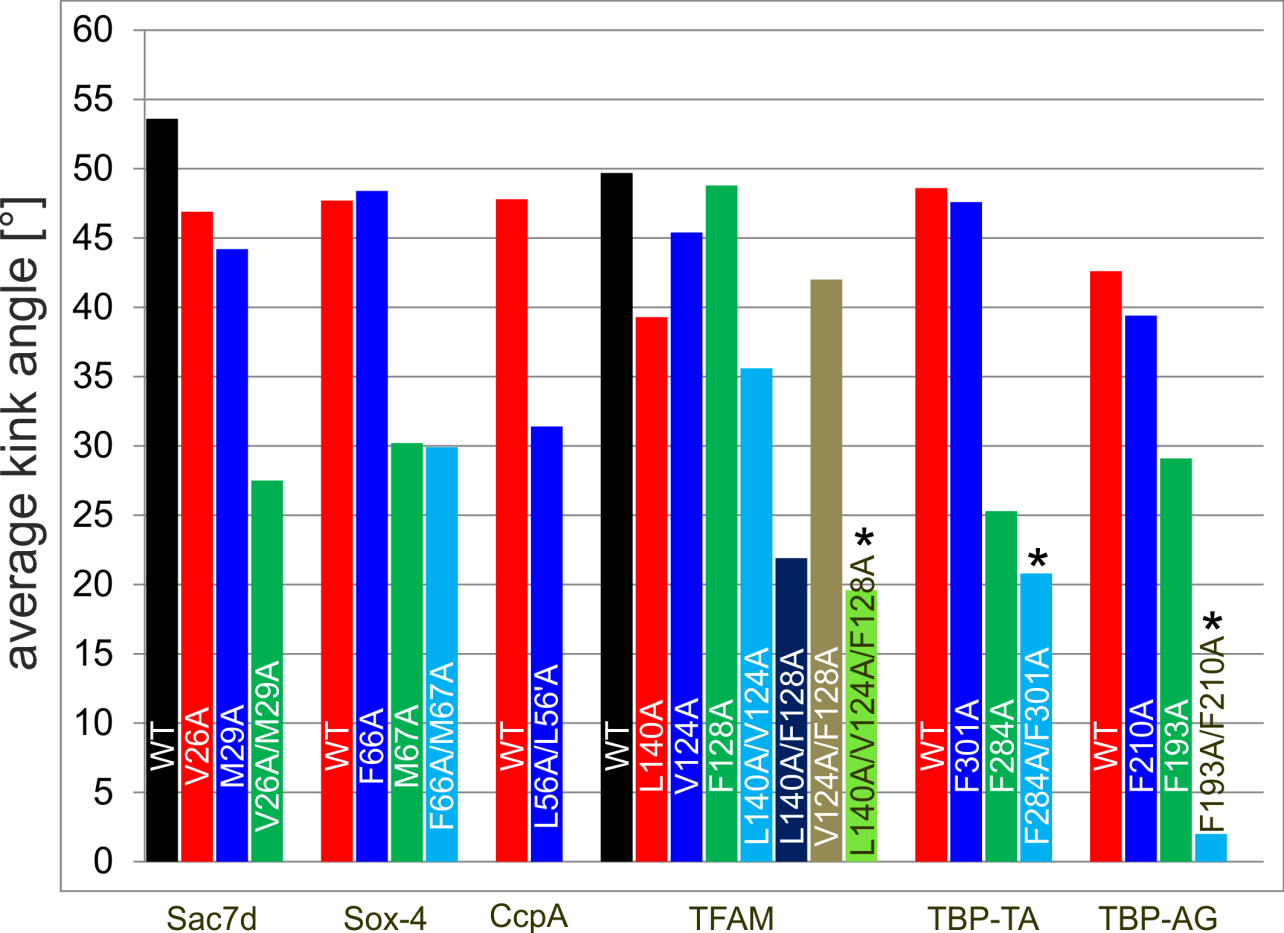
Summary of average kink angles. Summary of average kink angles for MD simulations of all studied complexes, sorted by proteins. The two kinks induced by the TBP protein at a TA and an AG base step were analysed separately (TBP-TA, TBP-AG). Bars marked by an asterisk correspond to conformationally instable systems (see text for details).

In the analyses presented above, the effects resulting from the mutation of intercalating residues were investigated for each protein-DNA complex individually. To allow a comparison between different systems the average kink angles observed in all five different systems are compiled in Fig 10. The effects caused by the mutation of one or multiple inserting residues can be broadly categorized in three groups (a) Mutations having only a small effect on the kink angle, (b) Mutations resulting in stable conformations with reduced kink angles, (c) Mutations causing conformational instability. These groups are described in more detail below:

The first group of variants exhibits a high conformational stability in the simulations and retains large kink angles of more than 35°. These properties are mainly observed for single mutants (e.g. Sac7d(V24A) or Sox-4(F66A)) but also for some double mutants (e.g. TFAM(V124A/F128A)). The small effect of the respective exchanges indicates that the mutant proteins still retain effective mechanisms to stabilize the kink in the DNA.

The second group of mutations resulted in stable conformations with reduced kink angles. One common feature of this group of mutants is that they relax within the first few nanoseconds of simulation time to a reduced kink angle of ~20–35°. The fast relaxation suggests a low energy barrier for the transition, which is also in line with the observation that a strong kink in Sox-4 can readily be re-established by an A67M exchange introduced in the relaxed mutant structure (Fig 5D). For the Sac7d system, the magnitude of the kink obtained from the simulation of the double mutant is well in agreement with the experimental value observed [29] suggesting that the relaxation process can be adequately monitored by the simulation times of the present study. It is interesting to note that none of the mutants belonging to this group shows further relaxation to smaller kink angles. Since all intercalating residues were replaced by alanine, one might speculate that this residual kink reflects a residual intercalation of the alanine sidechain methyl group. However, the additional A87G and M67G variants investigated for Sox-4 did not result in a relaxation towards smaller kink angles (S2 Fig). This indicates that the residual kink angles of ~30° can at least not primarily attributed to the presence of an alanine sidechain, but might rather result from the overall structural features of the binding proteins. This is in line with the observation that kinking does not necessarily require sidechain intercalation as exemplified by the CRP, EcoRV and Cre systems analyzed in Fig 2. We also assessed whether the smaller kink angle observed after mutation of the intercalating residues is also accompanied by an enhanced DNA flexibility. For that purpose the kink angles observed over the simulation time were plotted as histograms (S5 Fig). These diagrams show that even in case of reduced average kink angles the width of the angle distribution is rather similar to that observed for the wildtype. This demonstrates that intercalation is important to induce strong kinks but does not significantly reduce DNA flexibility.

We also identified a third group of mutants, which cause conformational instability. The respective systems do not relax to a defined kink angle but instead sample multiple distinct conformations over the simulation time (bars marked by asterisks in Fig 10). In two of the variants (TBP-TA(F284A/F301A) and TFAM(L140A/V124A/F128A) the position of the kink becomes shifted to the adjacent base step indicating a certain degree of conformational heterogeneity (Fig 9, S4 Fig). However, the conformational changes that occur upon the respective mutations are not adequately sampled in the simulation times of the present study. Significantly longer simulations will be required in future to assess whether these larger fluctuations indicate the onset of a dissociation process or rather reflect a broader conformational landscape with distinct kink angles.

The analysis above revealed that mutations can basically have three different types of effects on the magnitude and stability of the kinks. In a subsequent analysis, we also assessed the different possibilities of interdependence between multiple mutations within the same system and identified at least two different situations:

(i) Systems with a distinct role of the intercalating sidechains (e.g. Sox-4 and TBP). In these complexes, one of the residues proved to be essential for kink formation (M67 of Sox-4, Phe(loop) of TBP), whereas the second residue has only a very small effect on the magnitude of the kink (F66 of Sox-4, Phe(sheet) of TBP) (Figs 5 and 8).

(ii) Systems with a redundant role of the intercalating sidechains (Sac7d). In case of Sac7d either V26 or M29 can be mutated without causing a significant decrease of the kink angle (Fig 4). However, the low kink angle in the V26A/M29A double mutant indicates that at least one of the residues is required to maintain a strong kink.

For the TFAM system, which contains one intercalating and two partially intercalating residues, we observed a mixture of situations (i) and (ii) described above. Like in (i), there is one residue (V124), which has only a small effect on the magnitude of the kink. However, there is no single essential residue for kink formation, and L140 and F128 exhibit a rather redundant role, which is similar to case (ii) above. This redundant role can be seen from the fact that either L140 or F128 can be mutated without causing a significant decrease of the kink angle (Fig 9). However, the low kink angle in the L140A/F128A double mutant indicates that at least one of the residues is required to maintain a strong kink.

In summary, the present work has demonstrated that intercalating DNA residues play distinct roles for kink formation in different systems. In case of multiple inserted residues, not all of them are necessarily required for kink formation. Mutation of intercalating residues causes a broad spectrum of effects ranging from unaltered over reduced kink angles to a conformational instability of the kinked site. Molecular dynamics can help to identify the role of individual inserted residues for kinking, which is not readily apparent from an inspection of the static structures. This information should be helpful for a more detailed understanding of protein-DNA interactions.

## Supporting information

S1 Table PDB codes of all hits obtained from the database search shown in Fig 1. (PDF)

S1 Fig Time course of roll angles for Sox-4 wildtype and alanine mutants. Time course of the roll angles for all base steps over the MD simulation. The individual bases are plotted as y-axis starting with the 5’-end at the bottom. The vertical black line denotes the boundary between the two independent 250-ns MD simulations performed for each system, which are presented in a single panel. Colors of boxes match line colors of average roll angle plots of corresponding systems in Fig 5C.
(TIF)

S2 Fig Time course of roll angles for Sox-4 glycine mutants. Time course of the roll angles for all base steps over the MD simulation. The vertical black line denotes the boundary between the two independent 250-ns MD simulations performed for each system, which are presented in a single panel. (A) Simulation of a M67G variant representing the removal of an intercalating residue at the TT base step. (B) Simulation of an A87G variant intended to probe the role of A87 for kink formation at a TA base step.
(TIF)

S3 Fig Time course of roll angles for CcpA. Time course of the roll angles for all base steps over the MD simulation. The vertical black line denotes the boundary between the two independent MD simulations performed for each system, which are presented in a single panel. Colors of boxes match line colors of average roll angle plots of corresponding systems in Fig 6C.
(TIF)

S4 Fig Time course of roll angles for TFAM (Box-2). Time course of the roll angles for all base steps over the MD simulation. The vertical black line denotes the boundary between the two independent 250-ns MD simulations performed for each system, which are presented in a single panel.
(TIF)

S5 Fig Histograms of roll angles at the kinked positions. Exemplary histogram plots for CcpA (left panels) and TA-kink of TBP (right panels) for wildtypes (upper panels) and conformationally stable mutants with reduced kink angles (lower panels). The last panel is named according to mutation of the residue relevant for TA-kink (F284A), while the data originates from mutant F193A/F284A simulations. The solid line shows fit of data to normal distribution (gauss function fit: y = a*e^b(x-c)2^).
(TIF)
