# Supplementary material for: Probing the role of intercalating protein sidechains for kink formation in DNA
Source: PLoS One. 2018 Feb 12;13(2):e0192605. doi: 10.1371/journal.pone.0192605 (PMC5809078; doi:10.1371/journal.pone.0192605)
Supplement: S1 Table — (PDF) [file pone.0192605.s001.pdf]

CAP/CRP	1cgp, 1j59, 1lb2, 1o3q, 1o3r, 1o3s, 1o3t, 1run, 1ruo, 1zrc, 1zrd, 1zre, 1zrf, 2cgp, 3mzh, 3n4m
CBC	4g92
CcpA	3oqm, 3oqn, 3oqo, 1rzr
Cren7	3kxt, 3lwh, 3lwi,
cre-recombinase	1nzb, 1q3u, 2hof, 2hoi, 3c28, 3c29, 4crx ,5crx
EcoRV	1az0, 1b94, 1b95, 1b96, 1b97, 1bgb, 1bss, 1eoo, 1eop, 1rv5, 1rva, 1rvb, 1suz, 1sx8, 2b0d, 2ge5
Hbb	2np2
HmgD	1qrv
Lac	1efa, 1jwl, 1lbg
NF-gamma	4awl
Sac7d	1azp, 1azq, 1bnz, 1ca5, 1wd0
Sox	1gt0, 3f27, 3u2b, 4euw
TBP	1c9b, 1cdw, 1jfi, 1nvp, 1tgh, 1vol, 4roc, 4rod, 4roe
TFAM	3tmm, 3tq6, 4nnu, 4nod
Topoisomerase	3foe, 3fof, 3k9f, 3ksa, 3ksb, 3ltn, 3rad, 3rae, 3raf, 4koe, 4kpe, 4kpf
