# Supplementary figures and images for: Probing the role of intercalating protein sidechains for kink formation in DNA
Source: PLoS One. 2018 Feb 12;13(2):e0192605. doi: 10.1371/journal.pone.0192605 (PMC5809078; doi:10.1371/journal.pone.0192605)

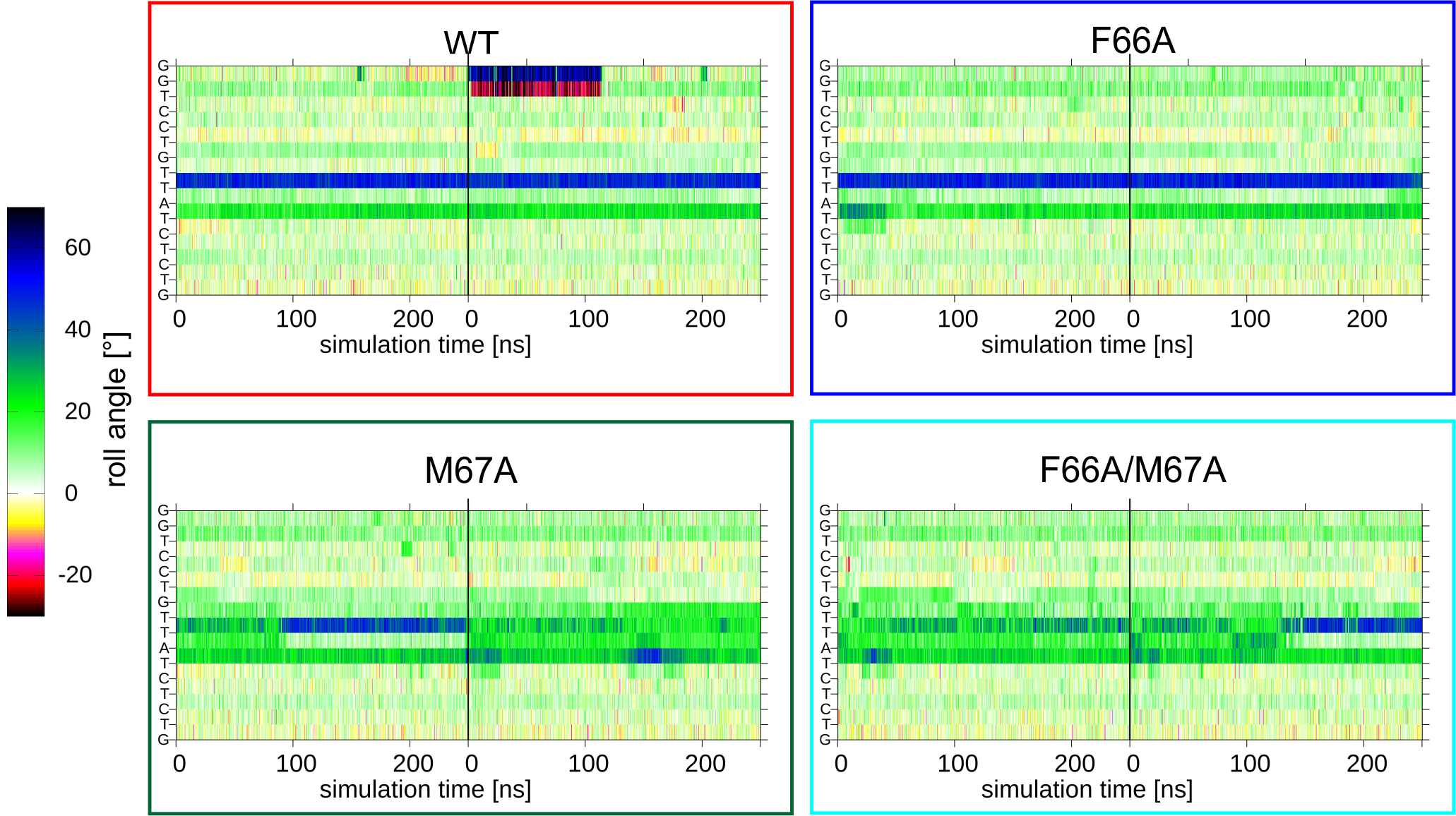

Supplement: S1 Fig — Time course of the roll angles for all base steps over the MD simulation. The individual bases are plotted as y-axis starting with the 5’-end at the bottom. The vertical black line denotes the boundary between the two independent 250-ns MD simulations performed for each system, which are presented in a single panel. Colors of boxes match line colors of average roll angle plots of corresponding systems in Fig 5C. (TIF) [file pone.0192605.s002.tif]

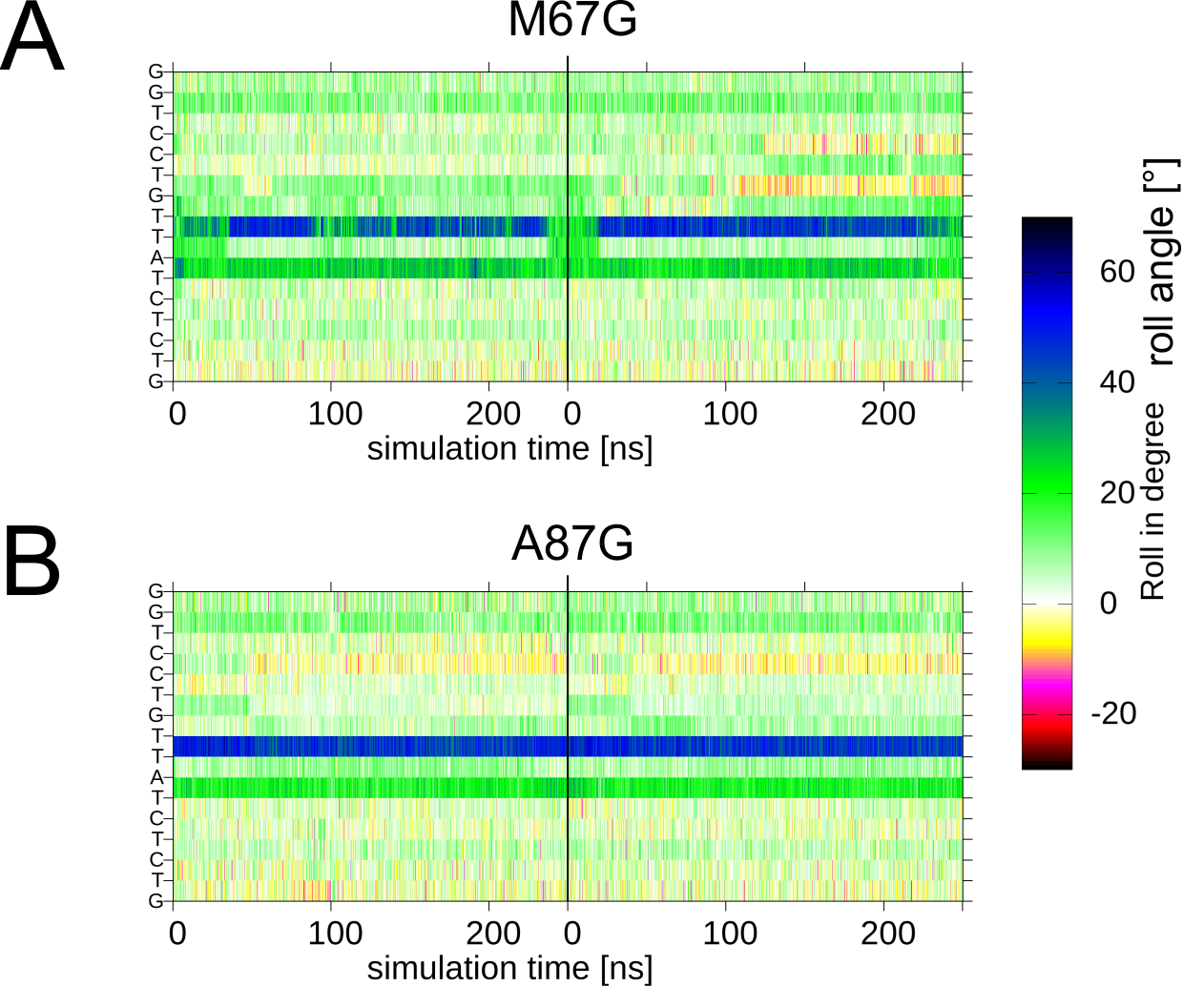

Supplement: S2 Fig — Time course of the roll angles for all base steps over the MD simulation. The vertical black line denotes the boundary between the two independent 250-ns MD simulations performed for each system, which are presented in a single panel. (A) Simulation of a M67G variant representing the removal of an intercalating residue at the TT base step. (B) Simulation of an A87G variant intended to probe the role of A87 for kink formation at a TA base step. (TIF) [file pone.0192605.s003.tif]

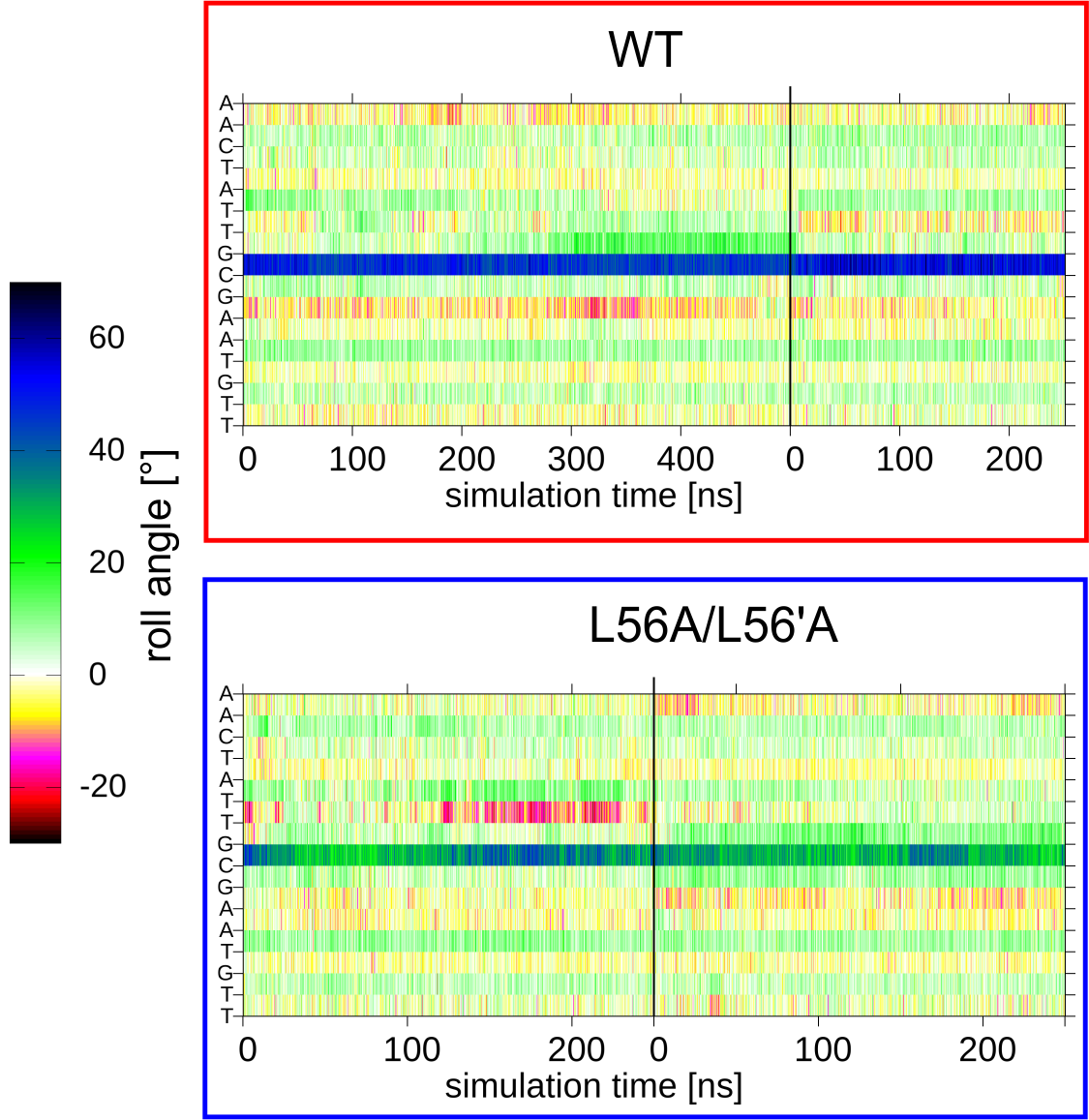

Supplement: S3 Fig — Time course of the roll angles for all base steps over the MD simulation. The vertical black line denotes the boundary between the two independent MD simulations performed for each system, which are presented in a single panel. Colors of boxes match line colors of average roll angle plots of corresponding systems in Fig 6C. (TIF) [file pone.0192605.s004.tif]

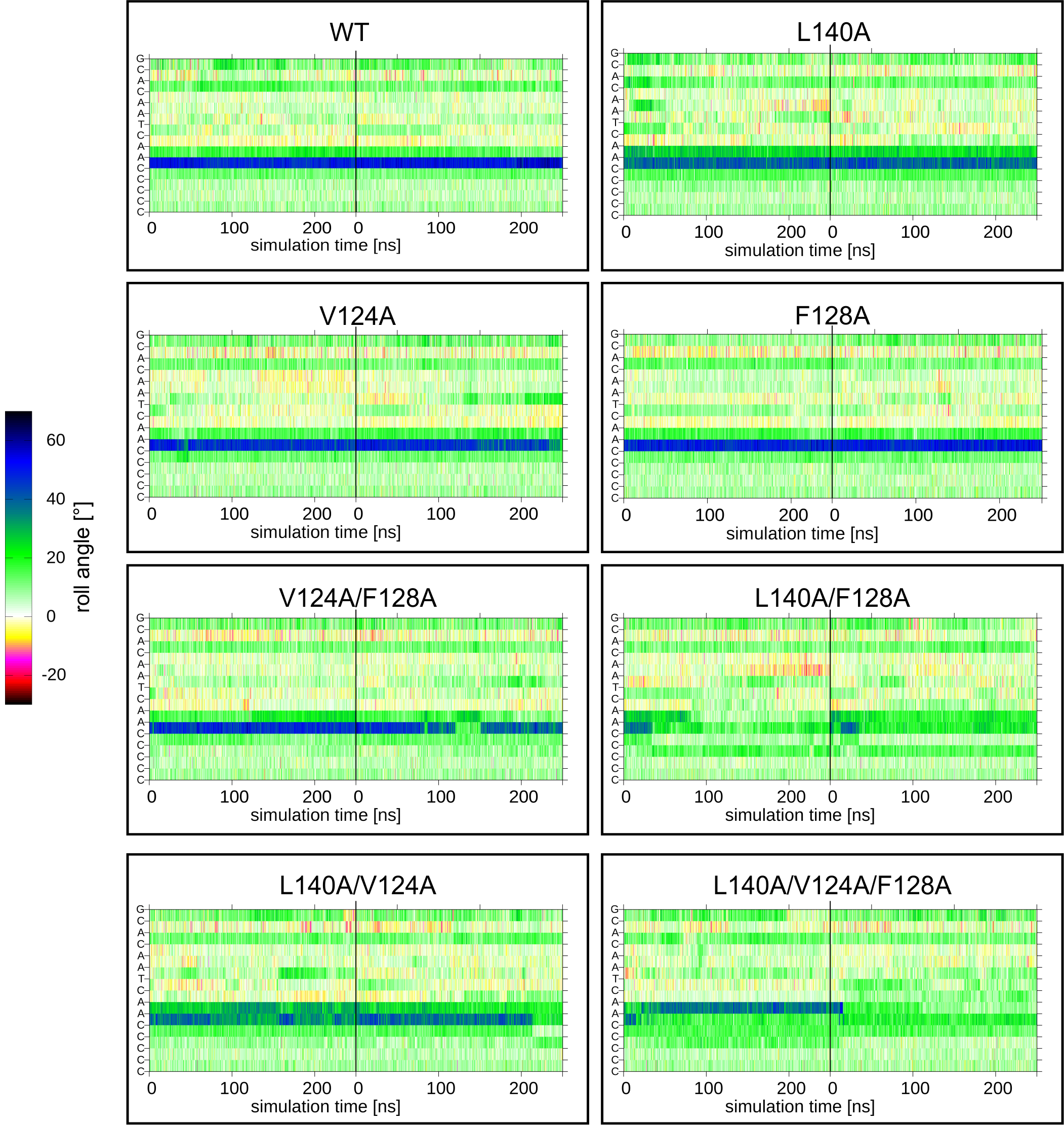

Supplement: S4 Fig — Time course of the roll angles for all base steps over the MD simulation. The vertical black line denotes the boundary between the two independent 250-ns MD simulations performed for each system, which are presented in a single panel. (TIF) [file pone.0192605.s005.tif]

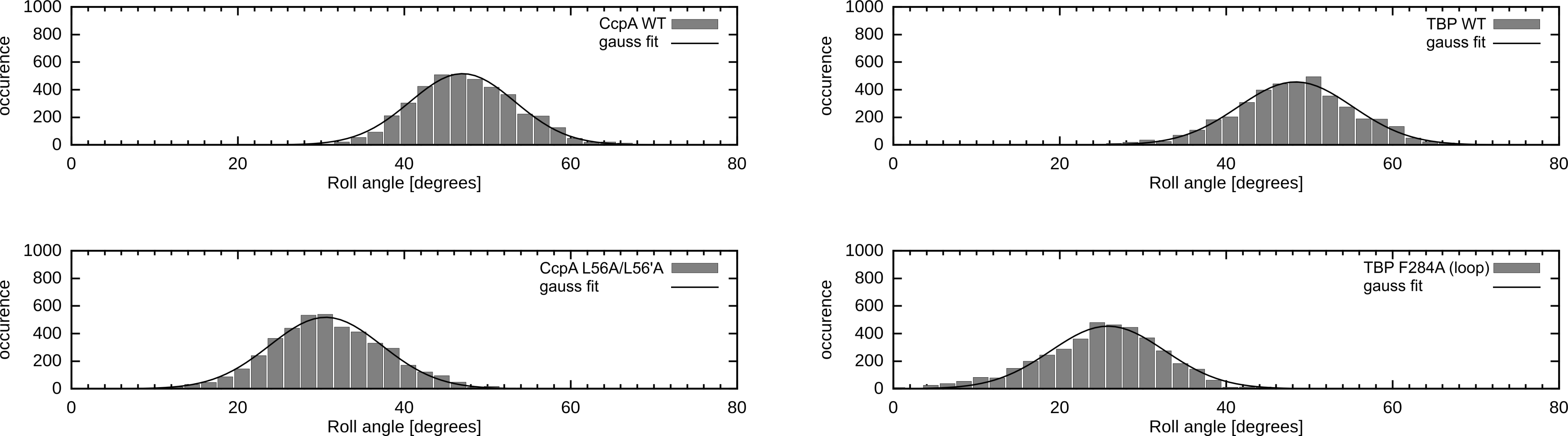

Supplement: S5 Fig — Exemplary histogram plots for CcpA (left panels) and TA-kink of TBP (right panels) for wildtypes (upper panels) and conformationally stable mutants with reduced kink angles (lower panels). The last panel is named according to mutation of the residue relevant for TA-kink (F284A), while the data originates from mutant F193A/F284A simulations. The solid line shows fit of data to normal distribution (gauss function fit: y = a*eb(x-c)2). (TIF) [file pone.0192605.s006.tif]
